# Dynamics of a Large-Scale Spiking Neural Network with Quadratic Integrate-and-Fire Neurons

**DOI:** 10.1155/2021/6623926

**Published:** 2021-02-23

**Authors:** Weijie Ye

**Affiliations:** ^1^School of Statistics and Mathematics, Guangdong University of Finance and Economics, Guangzhou 510320, China; ^2^Big data and Educational Statistics Application Laboratory, Guangdong University of Finance and Economics, Guangzhou 510320, China

## Abstract

Since the high dimension and complexity of the large-scale spiking neural network, it is difficult to research the network dynamics. In recent decades, the mean-field approximation has been a useful method to reduce the dimension of the network. In this study, we construct a large-scale spiking neural network with quadratic integrate-and-fire neurons and reduce it to a mean-field model to research the network dynamics. We find that the activity of the mean-field model is consistent with the network activity. Based on this agreement, a two-parameter bifurcation analysis is performed on the mean-field model to understand the network dynamics. The bifurcation scenario indicates that the network model has the quiescence state, the steady state with a relatively high firing rate, and the synchronization state which correspond to the stable node, stable focus, and stable limit cycle of the system, respectively. There exist several stable limit cycles with different periods, so we can observe the synchronization states with different periods. Additionally, the model shows bistability in some regions of the bifurcation diagram which suggests that two different activities coexist in the network. The mechanisms that how these states switch are also indicated by the bifurcation curves.

## 1. Introduction

Neurons and neural circuits can be modelled as nonlinear dynamical systems to simulate various brain activities. The dynamics of these dynamical systems provide theoretical insight into the biological mechanisms of brain functions. In recent decades, lots of neuron models have been intensively studied, and their equilibria and bifurcations are used to explain the formation and transition modes of the neuronal firing patterns [1–6]. However, because of the high dimension and complexity, the large-scale network models which consist of many neurons and synapses are difficult to study by dynamical methods [7]. Since the work of Wilson and Cowan [8], the mean-field approximation has become a common method to reduce the network dynamics to the mean firing rate, constructing a low-dimensional mean-field model. This kind of reduced model has been proven to be useful for us to understand the dynamical mechanisms of various underlying functions. Brunel et al. derived the mean-field approximation of the network with interacting excitatory and inhibitory neurons to study the synchronization of the neurons. The dynamics of the model exhibited different synchronization states, while the rhythmic transitions between the synchronization states were caused by the bifurcations of equilibria [9]. Besides, Brunel and Wang built a working memory network and reduced it to a mean-field model to research the computational mechanisms of how the brain stored the information temporarily [10]. They found that working memory was based on the bistability of the network where one stable state represents the memory activity and the other stable state denotes the spontaneous activity. Once an external stimulus input, the network changed from the spontaneous activity to the ongoing memory activity, indicating that the information of the stimulus has been remembered. This kind of bistability dynamics has also been observed in the decision-making function. Wong and Wang proposed a simplified approach to obtain the mean-field model from a decision-making network model which mimicked the decision-making process within two alternative choices [11, 12]. The decision-making process depended on the system tending towards one of the attractors for the two alternative choices separated by an unstable saddle in the decision space. Besides, different networks in visual processing [13–16], neural disease [17–19], and movement [20] have also been researched by mean-field dynamics.

In recent, Montbrió et al. proposed a new approach to reduce the large-scale neural network of quadratic integrate-and-fire neurons (QIF) as an exact mean-field model [21]. They found out that the QIF network model could be derived as a two-dimensional dynamical system for the firing rate and the mean membrane potential under the Lorentzian ansatz [22, 23]. The Lorentzian ansatz gave the distribution of the neurons' membrane potentials, so the continuity equation of the QIF network could be solved exactly, making the network possible to be theoretically analysed. Based on this work, Ratas et al. improved this model by considering more complicated synaptic dynamics so that the network with sophisticated synapses can also be reduced [24]. One of the characteristic properties of these mean-field models is the ability to exactly describing the network dynamics but not approximately reduce the network. Thus, the mean-field model derived by these methods can obtain the exact dynamical mechanism of the network models [25–28].

In this work, we construct a large-scale spiking neural network of quadratic integrate-and-fire neurons and reduce it to an exact mean-field model to investigate the network dynamics.

## 2. Methods

### 2.1. The Network Model

We consider a network model with an excitatory population and an inhibitory population. The total number of neurons in each population is 20000. Each neuron is modelled as the quadratic integrate-and-fire model which is described as

(1) $$\begin{array}{c} \frac{{\text{d}V}_{\text{k}}}{\text{d}t}=V_{k}^{2}+\eta +I_{\mathrm{ext}}-I_{k}^{\text{syn}}, \end{array}$$

where *V*_*k*_ represents the membrane potential and the index *k* can be *e* or *i* which marks the neuron in excitatory population or inhibitory population, respectively. *η* corresponds to a constant external current which follows a Lorentzian distribution *g*(*η*) of the mean $\bar{\eta }$ and half-width ∆, while *I*_ext_ is the external stimulus. Once the membrane potential exceeds the threshold  *V*_peak_, the neuron is considered to produce a spike and its voltage is reset to the value  *V*_reset_. To reduce the network analytically, *V*_peak_ and  *V*_reset_ are set to infinity, i.e., *V*_peak_ = −*V*_reset_ [29]. *I*_*k*_^syn^ is the total synaptic input which obeys

(2) $$\begin{array}{c} I_{k}^{\text{syn}}=I_{k}^{\text{exc}}+I_{k}^{\text{inh}}=J_{e}\left(V_{k}-E_{e}\right)S_{e}\left(t\right)+J_{i}\left(V_{k}-E_{i}\right)S_{i}\left(t\right)=J_{e}\left(V_{k}-E_{e}\right)\frac{1}{N}\overset{N}{\underset{l}{\sum }}H\left(V_{l}-V_{th}\right)+J_{i}\left(V_{k}-E_{i}\right)\frac{1}{N}\overset{N}{\underset{l}{\sum }}H\left(V_{l}-V_{th}\right), \end{array}$$

where *J*_*e*_ and *J*_*i*_ are excitatory and inhibitory synaptic conductance, *E*_*e*_ and *E*_*i*_ give excitatory and inhibitory reversal potential, while *S*_*k*_(*t*) are synapse activation variables which are simplified from a kind of alpha synapse function [24]. From Eq. (2), we can see that neurons in the excitatory population have recurrent excitatory synapses and inhibitory synaptic connections from the inhibitory population, while neurons in the inhibitory population receive recurrent inhibitory input and the excitatory synaptic input from the excitatory population.

### 2.2. The Mean-Field Model

In the  *N*⟶∞ limit, we can use continuous density functions *ρ*_*k*_(*V* | *η*, *t*) to describe the macroscopic state of each population in the system (1). The product *ρ*_*k*_(*V* | *η*, *t*) *dV* indicates the fraction of neurons with membrane potential between *V* and *V* + *dV*. Thus, under the conservation law of the neuron amount, the density functions *ρ*_*k*_(*V* | *η*, *t*) satisfy the continuity equations [9, 30, 31]:

(3) $$\begin{array}{c} \frac{\partial }{\partial \text{t}}\rho_{\text{k}}=-\frac{\partial }{\partial {\text{V}}_{\text{k}}}\left[\rho_{\text{k}}\left({\text{V}}_{\text{k}}^{2}+\eta +{\text{I}}_{\mathrm{ext}}+{\text{I}}_{\text{k}}^{\text{syn}}\right)\right]. \end{array}$$

With the assumption that the solutions of Eq. (3) generically converge to a Lorentzian-shaped function in any initial conditions, the density functions *ρ*_*k*_(*V* | *η*, *t*) can be expressed by following formula [21, 32–34]:

(4) $$\begin{array}{c} \rho_{k}\left(V\;|\;\eta ,t\right)=\frac{1}{\pi }\frac{x_{k}\left(\eta ,t\right)}{{\left[V-y_{k}\left(\eta ,t\right)\right]}^{2}+x_{k}^{2}\left(\eta ,t\right)\;}. \end{array}$$

This assumption makes it possible to solve the continuity equations Eq. (3) derived from the quadratic integrate-and-fire model.  *x*_*k*_(*η*, *t*)  and *y*_*k*_(*η*, *t*) define the half-width and the center of the distribution. These two parameters are closely related to the firing rate and mean membrane potential. With the fixing *η* and *t* , the firing rate of a population is calculated as the fraction of neurons which exceed the *V*_*peak*_, i.e., $r_{k}\left(\eta ,t\right)=\rho_{k}\left(V⟶\infty |\eta ,t\right)\dot{V}\left(V⟶\infty |\eta ,t\right)=x_{k}\left(\eta ,t\right)/\pi$. In cooperating with Eq. (1) and (4), and by integrating over *η* , we have

(5) $$\begin{array}{c} r_{k}\left(t\right)=\frac{1}{\pi }\int_{-\infty }^{+\infty }x_{k}\left(\eta ,t\right)g\left(\eta \right)d\eta . \end{array}$$

Besides, the center of the distribution of membrane potentials *y*_*k*_(*η*, *t*)  can be easily identified with the mean membrane potential which is given by *y*_*k*_(*η*, *t*) = p.v.∫_−∞_^+∞^*Vρ*_*k*_(*V* | *η*, *t*)*dV*. The result is the Cauchy principal value defined as $\text{p}.\text{v}.\int_{-\infty }^{+\infty }f\left(x\right)dx=\underset{R\to \infty }{\lim}\int_{-R}^{+R}f\left(x\right)dx$. Thus, the total mean membrane potential is

(6) $$\begin{array}{c} v_{k}\left(t\right)=\int_{-\infty }^{+\infty }y_{k}\left(\eta ,t\right)g\left(\eta \right)d\eta . \end{array}$$

Substituting Eq. (4) into the continuity equations Eq. (3), we can obtain

(7) $$\begin{array}{c} {\dot{w}}_{k}\left(\eta ,t\right)=i\left\{\eta -w_{k}^{2}\left(\eta ,t\right)+J_{e}\left[iw_{k}\left(\eta ,t\right)+E_{e}\right]S_{e}\left(t\right)+J_{i}\left[iw_{k}\left(\eta ,t\right)+E_{i}\right]S_{i}\left(t\right)\;\right\}, \end{array}$$

where *w*_*k*_(*η*, *t*) ≡ *x*_*k*_(*η*, *t*) + *iy*_*k*_(*η*, *t*). In this process, the synaptic activation variables *S*_*k*_(*t*) should be replaced by [24].

(8) $$\begin{array}{c} S_{k}\left(t\right)=\int_{-\infty }^{+\infty }g\left(\eta \right)\int_{-\infty }^{+\infty }\rho_{k}\left(V\;|\;\eta ,t\right)H\left(V-V_{th}\right)dVd\eta . \end{array}$$

For solving these equations, the distribution *g*(*η*) is assumed to the Lorentzian distribution with center $\bar{\eta }$ and half-width Δ:

(9) $$\begin{array}{c} g\;\left(\eta \right)=\frac{1}{\pi }\frac{\Delta }{{\left[\eta -\bar{\eta }\right]}^{2}+\Delta^{2}\;}. \end{array}$$

This assumption makes Eq. (5), (6), and (8) evaluated closing the integral contour in the complex *η* plane [21, 24]. In the lower half *η* plane, these integrals only rely on the pole $\eta =\bar{\eta }-i\Delta$ of g(*η*), and we have

(10) $$\begin{array}{c} \pi r_{k}\left(t\right)+iv_{k}\left(t\right)=w_{k}\left(\bar{\eta }-i\Delta ,t\right),\\ S_{k}\left(t\right)=\frac{1}{\pi }\left\{\frac{\pi }{2}-\arctan\left[\frac{V_{th}-v_{k}\left(\text{t}\right)}{{\text{r}}_{k}\left(\text{t}\right)}\right]\right\}. \end{array}$$

By substituting these equations into Eq. (7) and separating the imaginary and real parts, we can obtain the following exact mean-field model:

(11) $$\begin{array}{c} \left\{\begin{array}{c} \frac{\text{d}{\text{r}}_{\text{e}}}{\text{dt}}=\frac{\Delta }{\pi }+2{\text{r}}_{\text{e}}{\text{v}}_{\text{e}}-{\text{J}}_{\text{e}}{\text{r}}_{\text{e}}{\text{S}}_{\text{e}}\left(\text{t}\right)\\ \frac{\text{d}{\text{v}}_{\text{e}}}{\text{dt}}=\bar{\eta }+{\text{v}}_{\text{e}}^{2}-\pi^{2}{\text{r}}_{\text{e}}^{2}-{\text{J}}_{\text{e}}\left({\text{v}}_{\text{e}}-{\text{E}}_{\text{e}}\right){\text{S}}_{\text{e}}\left(\text{t}\right)-{\text{J}}_{\text{i}}\left({\text{v}}_{\text{e}}-{\text{E}}_{\text{i}}\right){\text{S}}_{\text{i}}\left(\text{t}\right)+{\text{I}}_{\mathrm{ext}}\\ \frac{\text{d}{\text{r}}_{\text{i}}}{\text{dt}}=\frac{\Delta }{\pi }+2{\text{r}}_{\text{i}}{\text{v}}_{\text{i}}-{\text{J}}_{\text{i}}{\text{r}}_{\text{i}}{\text{S}}_{\text{i}}\left(\text{t}\right)\\ \frac{\text{d}{\text{v}}_{\text{i}}}{\text{dt}}=\bar{\eta }+{\text{v}}_{\text{i}}^{2}-\pi^{2}{\text{r}}_{\text{i}}^{2}-{\text{J}}_{\text{e}}\left({\text{v}}_{\text{i}}-{\text{E}}_{\text{e}}\right){\text{S}}_{\text{e}}\left(\text{t}\right)-{\text{J}}_{\text{i}}\left({\text{v}}_{\text{i}}-{\text{E}}_{\text{i}}\right){\text{S}}_{\text{i}}\left(\text{t}\right) \end{array}\right.. \end{array}$$

In the mean-field model and the original network model, the default parameters are *J*_*e*_ = 15, *J*_*i*_ = 8, *E*_*e*_ = 75, *E*_*i*_ = −75, $\bar{\eta }=-5$, Δ = 1, and *V*_*th*_ = 50. The simulation is performed in Python by using the four order Runge-Kutta algorithm with an integration time step of 0.01 ms. Bifurcation analysis is conducted in MATCONT.

## 3. Results

### 3.1. Activities of the Network Model and Mean-Field Model

**Figure 1:**
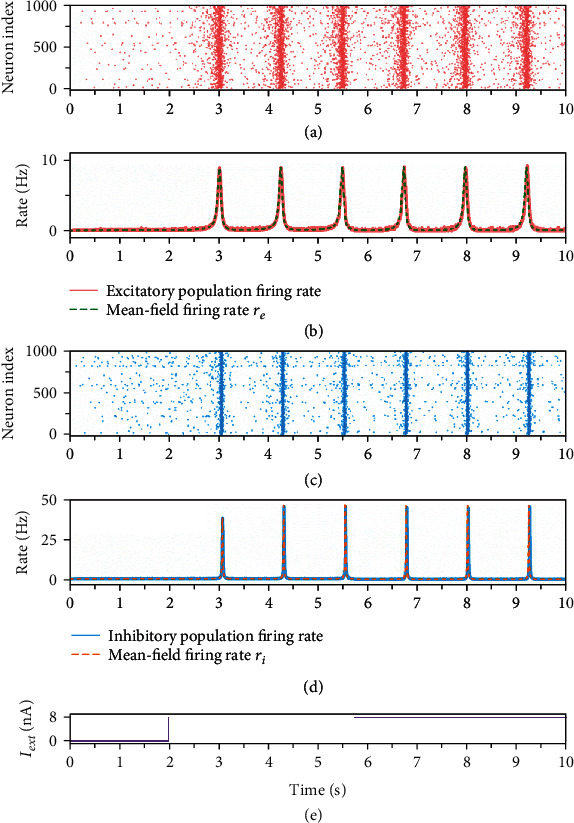
Activities of the network model and the mean-field model. (a) Raster plot of 1000 randomly selected neurons in the excitatory population. (b) The firing rate of the excitatory population (red solid) and the mean-field firing rate *r*_*e*_ (green dashed). (c) Raster plot of 1000 randomly selected neurons in the inhibitory population. (b) The firing rate of the inhibitory population (blue solid) and the mean-field firing rate *r*_*i*_ (orange dashed). (e) At *t* = 0, a current *I*_ext_ = 0 is applied to all excitatory neurons, and set to *I*_ext_ = 8 at *t* = 2.

For testing whether the activity of the mean-field model is consistent with the network model, both models receive the same external stimuli. As is shown in Figure 1(e), the external stimulus is set to *I*_ext_ = 0 at 0-2 s, and increases to *I*_ext_ = 8 at 2-10 s. When *I*_ext_ = 0, i.e., there is no external input, the excitatory population and inhibitory population of the network model both show the quiescence states which only exhibit the background activities (Figures 1(a) and 1(c)). At this time, the corresponding mean-field firing rates *r*_*e*_ and *r*_*i*_ are also at the quiescence states which are consistent with the network model firing rates (Figures 1(b) and 1(d)). After 2 s, the external stimulus is input to the excitatory population. As is shown in Figures 1(a) and 1(c), the external stimulus makes a large number of neurons in excitatory and inhibitory populations produce spikes in the same block of time, showing the synchronization states. It is noteworthy that synchronization defines a phenomenon that the action potentials occur closely together in time. As time increases, the spikes are periodically produced. As a result, the corresponding population firing rates exhibit periodical activities. In the synchronization state, the mean-field firing rates are also in agreement with the population firing rates (Figures 1(b) and 1(d)).

### 3.2. The Network Dynamics

The agreement between the network activity and mean-field activity enables us to study the network dynamics from the mean-field dynamics. In this section, we mainly use bifurcation analysis to research the dynamics of the mean-field model.

**Figure 2:**
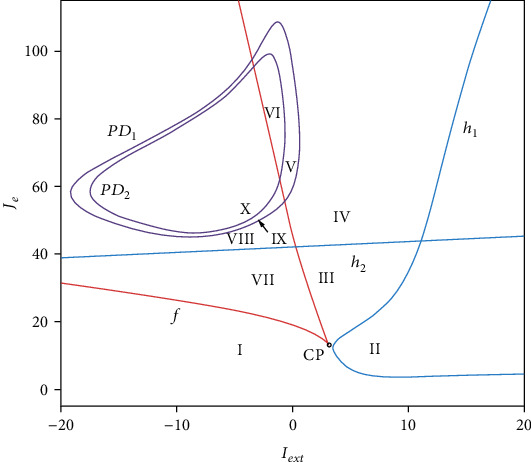
Two-parameter bifurcation diagram in the *I*_ext_-*J*_*e*_ plane. *f*: fold bifurcation curve (red); *h*_1_, *h*_2_: Hopf bifurcation curve (blue); *PD*_1_, *PD*_2_: period-doubling bifurcation curve (purple).

**Figure 3:**
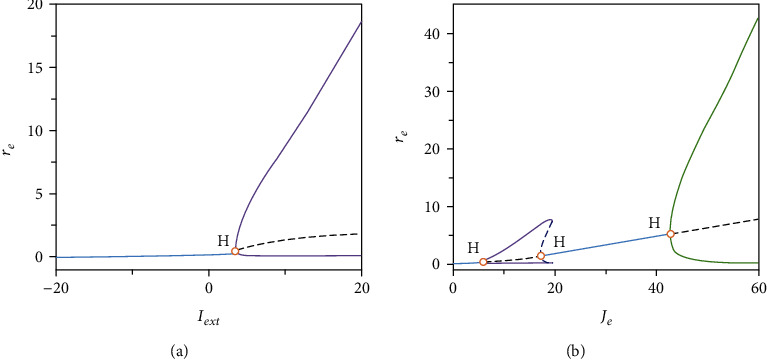
Two single-parameter bifurcation diagrams with respect to *I*_ext_ when *J*_*e*_ = 12 (a) and *J*_*e*_ when *I*_ext_ = 5 (b). The blue solid lines represent the stable equilibria, and the black dashed lines give the unstable equilibria. The purple solid lines and the green solid lines represent the maxima and minima of the stable limit cycles. The blue dashed lines identify the maxima and minima of the unstable limit cycles. The orange circles indicate the Hopf bifurcation points.

**Figure 4:**
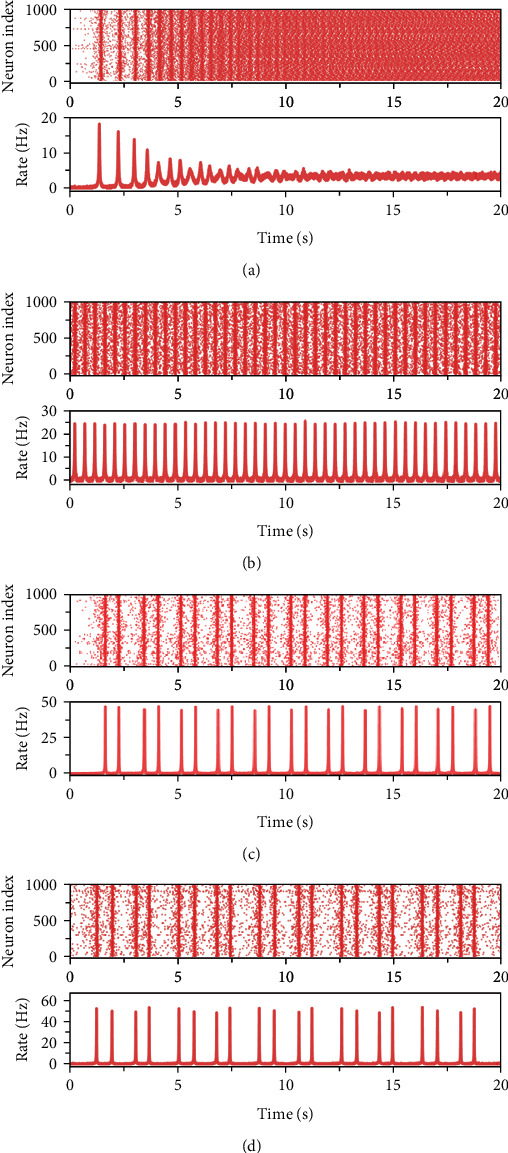
Activities of the network model when (*I*_ext_, *J*_*e*_) = (5, 30) (a), (*I*_ext_, *J*_*e*_) = (5, 50) (b), (*I*_ext_, *J*_*e*_) = (0, 70) (c), and (*I*_ext_, *J*_*e*_) = (−0.85, 75) (d). In each chart, the upper panel is the raster plot of 1000 randomly selected neurons in the excitatory population, the bottom panel gives the corresponding firing rate of the excitatory population.

In a dynamical system, a bifurcation is a sudden qualitative or topological change in behavior of the system caused by a small smooth change of some parameter values. Thus, we can understand how the network activities change from the bifurcations of the mean-field system.  Figure 2 provides an overall two-parameter bifurcation scenario in the *I*_ext_-*J*_*e*_ plane. The parameter plane is partitioned into 10 regions by the following bifurcation curves: *f* fold of equilibria (red); *h*_1_ and *h*_2_ Hopf (blue); *PD*_1_ and *PD*_2_ period-doubling (purple). We label these 10 regions as I-X. The network model in different regions exhibits distinct dynamics, and the transition between them can explain how the network activities change. The corresponding dynamics are described as follows:

1. In region I, the model exhibits monostability with a single stable equilibrium point (e.g., *I*_ext_ = 0, *J*_*e*_ = 15, 0-2 s in Figure 1). The single-parameter bifurcation chart in Figure 2 also indicates that this state is at a low population firing rate which corresponds to the quiescence state of the network model. It is common that a number of functional neural networks in brain are at a quiescence state. When the networks only receive the background input, they usually exhibit a low firing rate
2. Crossing *h*_1_ Hopf bifurcation curve makes the model appear stable limit cycle and the stable equilibrium turns into unstable equilibrium in region II (Figure 3(a)). The stable limit cycle induces the model to produce the firing rate spikes regularly, indicating the synchronization state of the network model (e.g., *I*_ext_ = 8, *J*_*e*_ = 15, 2-10 s in Figure 1)
3. Region III lays around the *f* fold curve, the *h*_1_ Hopf bifurcation curve, and the *h*_2_ Hopf bifurcation curve. There is one stable focus in this region (Figure 3(b)). As shown in Figure 4(a), the network in this state exhibits the synchronization state in the beginning. With time increases, the degree of synchronization weakens and the firing rate tends to a stable level which is higher than the quiescence state
4. As the parameters change from Region III to Region IV via *h*_2_ Hopf bifurcation curve, the stable focus turns into unstable and the stable limit cycle appears (Figure 3(b)). The activity of the network in this region oscillates with the same period which is similar to that in region II (Figure 4(a)). A large number of neurons are in the synchronization state
5. When crossing *PD*_1_ period-doubling curve from region IV, the period of the stable limit cycle becomes period-2. In this region, the excitatory population firing rate oscillates with two periods, exhibiting the bursting synchronization state (Figure 4(c))
6. In region VI, *PD*_2_ period-doubling bifurcation curve doublings the period of the stable limit cycle, making it turn into period-4. Thus, we can see that the excitatory neurons synchronize with four periods and the corresponding firing rate oscillates regularly with a series of the burst with four periods (Figure 4(d)).

The synchronization states of the network shown in (2), (4)–(6) have been considered as one of the mechanisms for neuronal signal transmission and coding. For example, synchronization can protect the neural information from noise [35]. In addition, synchronization has been associated with a large number of functions such as memory, attention, object recognition, and top-down modulation of sensory processes [36, 37]. However, abnormal synchronization will lead to several neural disorders, including epilepsy [38] and Parkinson's disease [39].

**Figure 5:**
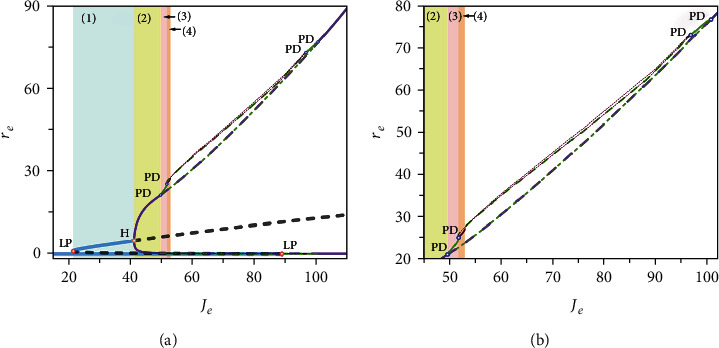
Two single-parameter bifurcation diagrams with respect to *J*_*e*_ when *I*_ext_ = −3. The chart (b) is the enlargement of the top-right part of the chart (a). The blue solid lines represent the stable equilibria, and the black dashed lines give the unstable equilibria. The purple solid lines and dashed lines represent the maxima and minima of the stable and unstable limit cycles with period-1, respectively. The green solid lines and dashed lines give the maxima and minima of the stable limit cycles with period-2, respectively. The red solid lines and dashed lines give the maxima and minima of the stable limit cycles with period-4, respectively. The orange, red, and purple circles indicate the Hopf, fold, and period-doubling bifurcation points, respectively. Regions (1), (2), (3), and (4) represent four different regions of bistability.

**Figure 6:**
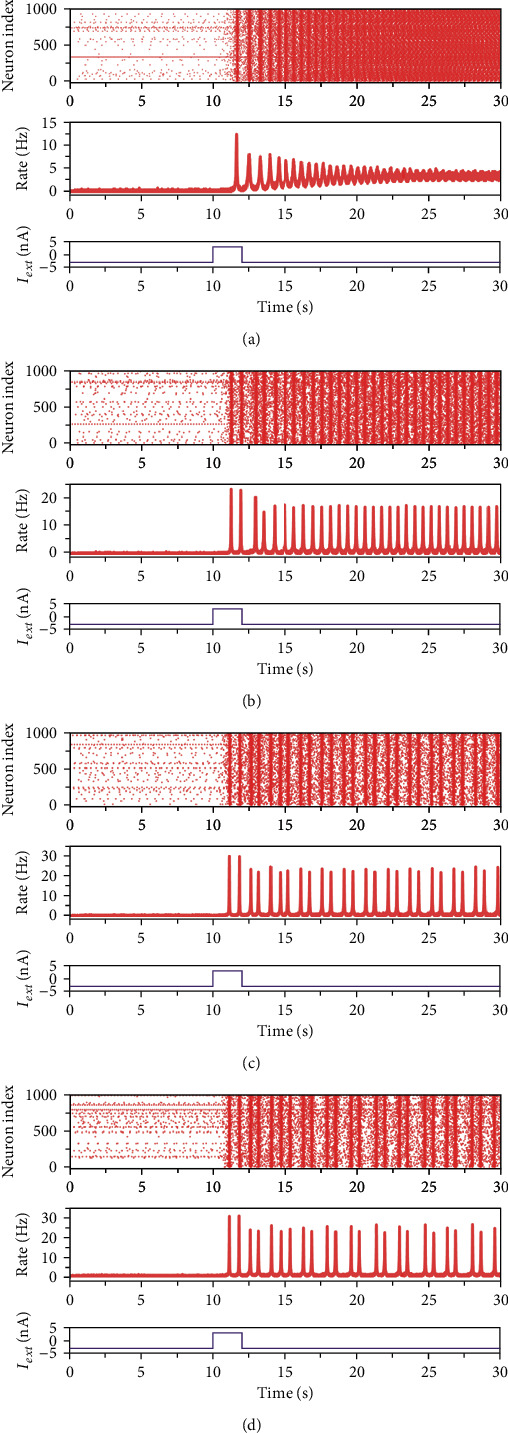
Activities of the network model when (*I*_ext_, *J*_*e*_) = (−3, 32) (a), (*I*_ext_, *J*_*e*_) = (−3, 45) (b), (*I*_ext_, *J*_*e*_) = (−3, 51) (c), and (*I*_ext_, *J*_*e*_) = (−3, 52.4) (d). In each chart, the upper panel is the raster plot of 1000 randomly selected neurons in the excitatory population, the middle panel identifies the corresponding firing rate of the excitatory population, and the bottom panel gives variation of the external stimulus which I_ext_ = 3 between 10 and 12 s, and *I*_ext_ = −3 at other time.

(7) Inside the LP curve, the network shows bistability. However, the coexistent stable equilibria are different in distinct parts. As it is shown in Figure 5(a), the blue area (1) of the single-parameter bifurcation diagram, which is located in the region VII, indicates that the solid lines above and under the dashed line correspond to the stable focus and stable node, respectively. Thus, we can know that the stable focus and stable node coexist in the region VII. This fact is demonstrated by the network activity. When *J*_*e*_ = 32, *I*_ext_ = −3, the excitatory population maintains the quiescence state of a low firing rate level which corresponds to the stable node (Figure 6(a)). Once a transient stimulus is applied at 10-12 s, the firing rate produces a spike. After 12 s, the stimulus returns to *I*_ext_ = −3, the firing rate does not recover to the quiescence state but tends to a steady firing rate at about 4.5 Hz which corresponds to the stable focus. Thus, two kinds of activities also coexist in the network model
(8) In regions VIII, IX, and X, the stable node and stable limit cycle with different periods coexist in the network. As an example, the stable node and stable limit cycle with period-1 coexist in the area (2) in Figure 5(a), the stable node and stable limit cycle with period-2 coexist in the area (3), and the stable node and stable limit cycle with period-4 coexist in the area (4). These facts indicate that the corresponding network activities also exhibit bistability. As illustrated in Figures 6(b)–6(d), the transient stimulus at 10-12 s makes the original steady quiescence state become regular oscillation with different periods in distinct regions (one period for region VIII, two periods for region IX, and four periods for region X).

Bistability in neural network induces the persistent activity after a transient stimulus vanishes. This kind of persistent activity underlies short-term information storage in brain functions such as working memory, motor control, and spatial navigation [40, 41].

**Figure 7:**
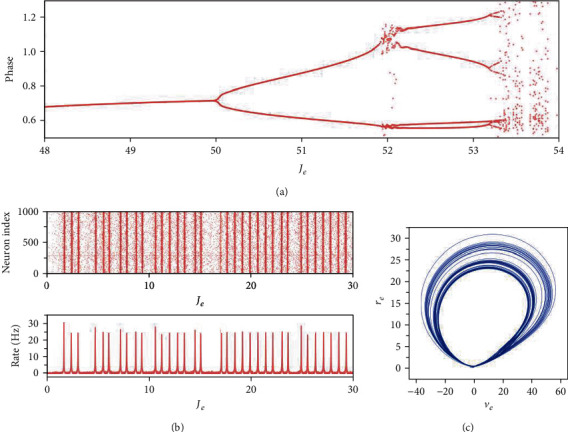
(a) The intervals between spikes of the firing rate with respect to *J*_*e*_. (b) Activities of the network model when (*I*_ext_, *J*_*e*_) = (−3, 53.5). The upper panel is the raster plot of 1000 randomly selected neurons in the excitatory population, the bottom panel gives the corresponding firing rate of the excitatory population. (c) The trajectory of the mean-field model in the (*v*_*e*_, *r*_*e*_) plane when (*I*_ext_, *J*_*e*_) = (−3, 53.5).

By fixing *I*_ext_ = −3 and changing *J*_*e*_ from 48 to 54, we calculate the intervals between spikes of the firing rate which represent the period of the firing rate. The transition of the phase of the synchronization state in regions VIII, IX, and X is also indicated by the change of period (Figure 7(a)). From the bifurcation diagram, we can see the period-doubling cascade of the phase. It is notable that this period-doubling cascade finally leads to chaos. The chaotic activities of the network are illustrated in Figures 7(b) and 7(c). Although the neurons exhibit synchronization, they synchronize without fixing phases. The firing rate also oscillates with no fixing periods. This chaotic behavior is also verified by the trajectory in the (*v*_*e*_, *r*_*e*_) plane of mean-field model.

## 4. Conclusions

In this paper, we constructed a large-scale spiking neural network with quadratic integrate-and-fire neurons and reduced to a mean-field model to research the network dynamics. First, the activity of the mean-field model was verified to be consistent with the activity of the network model. This agreement ensured that we could understand the network dynamics from the dynamical properties of the mean-field model. Thus, the two-parameter bifurcation analysis was performed on the mean-field model to research the network dynamics. From the bifurcation scenario, we could know that the network has various activities: the quiescence state, the steady state with a relatively high firing rate, and the synchronization state which corresponds to the stable node, stable focus, and stable limit cycle of the system, respectively. Notably, there existed several stable limit cycles with different periods, so the synchronization states with different periods of the network model could be observed. Additionally, bistability, i.e., two steady states coexisted, was observed in some regions of the two parameters plane. This result proved that two different activities coexisted in the network model that could switch from one to another by shortly perturbing some parameters. Finally, we found that the period-doubling cascade led to chaos. In this state, the network exhibited the chaotic synchronization state in which the neurons synchronized without fixing periods.

In future work, the current neural network and mean-field model can be used to research the neural disorders. For example, Parkinson's disease is characterized by the excessive synchronization in the microcircuit of the basal ganglia. The present models exhibit rich synchronization phenomena, so they can be adapted to the activities in basal ganglia or other brain areas to research the pathological mechanisms of neural disorders and the methods of controlling synchronization. Another direction for future research is cognition. Based on the bistability, the network model can simulate some cognitive functions such as working memory, attention to understand the corresponding dynamical mechanisms of cognition.

## Data Availability

No data were used to support this study.
